# Probing the Conformational Restraints of DNA Damage Recognition with β-L-Nucleotides

**DOI:** 10.3390/ijms25116006

**Published:** 2024-05-30

**Authors:** Anna V. Yudkina, Daria V. Kim, Timofey D. Zharkov, Dmitry O. Zharkov, Anton V. Endutkin

**Affiliations:** 1 Siberian Branch of the Russian Academy of Sciences Institute of Chemical Biology and Fundamental Medicine, 8 Lavrentieva Ave., 630090 Novosibirsk, Russia; ayudkina@niboch.nsc.ru (A.V.Y.); dkim@niboch.nsc.ru (D.V.K.); timazharkov74@gmail.com (T.D.Z.); 2 Department of Natural Sciences, Novosibirsk State University, 2 Pirogova St., 630090 Novosibirsk, Russia

**Keywords:** DNA polymerases, AP endonucleases, DNA glycosylases, DNA repair, translesion synthesis, β-L-nucleotides

## Abstract

The DNA building blocks 2′-deoxynucleotides are enantiomeric, with their natural β-D-configuration dictated by the sugar moiety. Their synthetic β-L-enantiomers (βLdNs) can be used to obtain L-DNA, which, when fully substituted, is resistant to nucleases and is finding use in many biosensing and nanotechnology applications. However, much less is known about the enzymatic recognition and processing of individual βLdNs embedded in D-DNA. Here, we address the template properties of βLdNs for several DNA polymerases and the ability of base excision repair enzymes to remove these modifications from DNA. The Klenow fragment was fully blocked by βLdNs, whereas DNA polymerase κ bypassed them in an error-free manner. Phage RB69 DNA polymerase and DNA polymerase β treated βLdNs as non-instructive but the latter enzyme shifted towards error-free incorporation on a gapped DNA substrate. DNA glycosylases and AP endonucleases did not process βLdNs. DNA glycosylases sensitive to the base opposite their cognate lesions also did not recognize βLdNs as a correct pairing partner. Nevertheless, when placed in a reporter plasmid, pyrimidine βLdNs were resistant to repair in human cells, whereas purine βLdNs appear to be partly repaired. Overall, βLdNs are unique modifications that are mostly non-instructive but have dual non-instructive/instructive properties in special cases.

## 1. Introduction

The versatility of synthetic oligonucleotides as important tools in basic research, biotechnology and biomedicine can hardly be overestimated. The well-studied properties and predictable behavior of oligonucleotides under a wide range of conditions have made it possible to rationally design complex nanoscale structures and devices, giving rise to the whole DNA nanotechnology field [1,2]. The most desirable applications of oligonucleotides are those taking place in complex biological environments, such as the cell’s interior. However, the half-life of exogenous natural nucleic acids under such conditions is only minutes to hours [3,4]. The main path to overcoming these limitations is the use of non-natural nucleotides, which should combine biological stability, low toxicity, low immunogenicity, and a predictable thermodynamic behavior.

**Figure 1:**
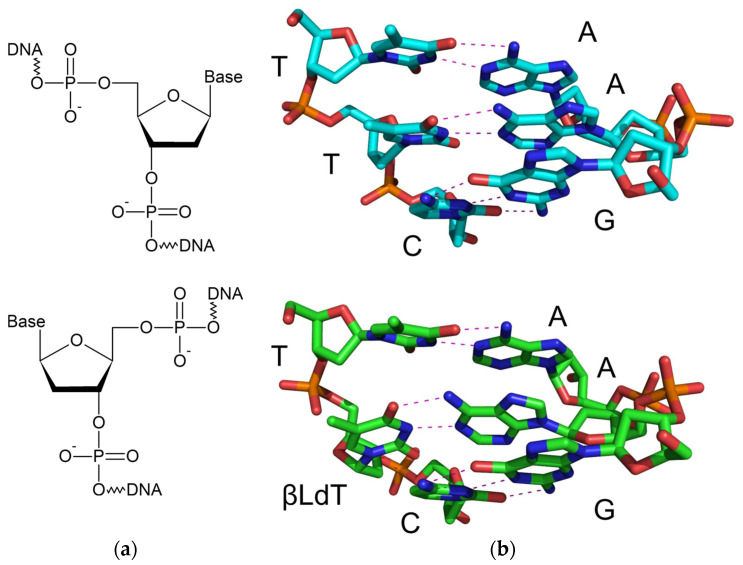
(**a**) Structures of normal β-D- (top) and mirror-image β-L-deoxynucleotides (bottom). (**b**) Structure of normal DNA (top, 5′-TTC-3′/5′-GAA-3′, PDB ID 355D [12]) and a hypothetical structure of 5′-T[βLdT]C-3′/5′-GAA-3′ maintaining Watson–Crick bonding in DNA (bottom; see Section 4 for the description of model building). Dashes indicate hydrogen bonds.

The enantiomers of natural nucleotides appropriately meet these criteria. Being chiral molecules, the natural D-isomers of nucleic acids have their mirror versions, L-DNA/RNA, containing β-L-(deoxy)ribose sugar moieties [5,6] (Figure 1a). L- and D-oligonucleotides exhibit identical physical properties, including the thermal stability of duplexes, hybridization kinetics, and solubility. The structures of L-DNA and D-DNA differ only in stereochemistry. This leads to differences in their interactions with chiral molecules: D-DNA selectively binds to its complementary D-DNA, forming a right-handed helix, while L-DNA specifically interacts with its complementary L-DNA, forming a left-handed helix. Due to this, L-DNA is highly orthogonal to the stereospecific environment of natural biological systems. Many enzymes that interact with D-DNA, including nucleases, typically do not recognize L-DNA. Besides the nuclease resistance, it has been demonstrated that L-DNA is non-toxic and non-immunogenic [7]. These unique properties of L-DNA and L/D-DNA hetero-oligomers have made them attractive for various biological applications, such as aptamers (Spiegelmers), molecular beacons, molecular tags, and drug nanocarriers [8,9,10,11]. The increased interest in technologies involving L-nucleic acids has been supported by the commercial availability of their synthetic building blocks over the past 20 years.

In the intracellular milieu, any foreign DNA will inevitably interact with the enzymatic systems carrying out DNA degradation, modification, replication, and repair. While the resistance of L-nucleic acids to various nucleases has been described [8,13], studies on their interactions with other DNA-dependent enzymes are scarce. It has been shown that T4 polynucleotide kinase, which has low specificity for the 5′-terminal nucleotide and tolerates even non-nucleotide modifications at this position, quite effectively phosphorylates the 5′-end of L-nucleic acids [14]. In the context of mining for anticancer and antiviral molecules among L-configured dideoxynucleosides it has been established that β-L-enantiomers of ddC, 5-fluoro-ddC, 1,3-dioxolane-cytidine, and 1,3-dioxolane-5-fluorocytidine can be phosphorylated by cellular kinases into the respective triphosphates [15], which can then serve as substrates for human DNA polymerases α, β, γ, δ, ε, and λ [16,17]. Since these L-derivatives lack a hydroxyl at the 3′ position, replication terminates after their incorporation. The resulting DNA with a blocked 3′-end can serve as a substrate for the exonuclease activity of apurinic/apyrimidinic endonuclease (APE1), and the removal of L-enantiomers is more efficient compared to normal nucleotides [18].

While promising technologies based on L-oligonucleotides and monomers have been developed, much less attention has been paid so far to the structural and functional properties of synthetic DNA containing single β-L-deoxynucleotides (βLdNs) embedded in otherwise regular D-DNA. NMR and melting data show that backbone adjustment allows such isolated βLdNs to engage in the Watson–Crick-type base pairing, destabilizing the duplex only moderately [19,20,21,22] (Figure 1b). It is interesting how such modifications could potentially interact with proteins involved in cellular systems dealing with non-canonical DNA, such as DNA repair, damage response and damage tolerance.

In the cellular context, any non-canonical nucleotide incorporated into DNA, save for natural modifications such as 5-methylcytosine or 5-hydroxymethylcytosine, is recognized as a lesion and subsequently removed. A wide range of non-bulky lesions are usually removed via the base excision repair (BER) pathway [23,24,25]. In the simplest case, the repair of such lesions is initiated by one of several DNA glycosylases, each specific for certain base modification types, which hydrolyze the *N*-glycosidic bond of the target nucleotide. The resulting apurinic/apyrimidinic site (AP site) is further processed by AP endonucleases (e.g., the aforementioned APE1 in human cells), which hydrolyze the 5′-phosphodiester bond of the AP site and expose a free 3′-OH end. The BER cycle is completed after a DNA polymerase incorporates a normal dNMP and a DNA ligase seals the remaining nick. Should the repair fail and allow the lesion to persist until replication, DNA polymerases could insert a dNMP opposite the non-canonical nucleotide, which often leads to a mutation but can prevent replication fork collapse and cell death. Replicative and repair polymerases vary greatly in their ability to carry out synthesis on damaged templates, and a special group, translesion DNA polymerases, are dedicated to error-prone lesion bypass.

Given the paucity of biochemical data, we set out to study the properties of all four βLdNs as template nucleotides for DNA polymerases representing the major polymerase families (A, B, X, and Y) and as lesions possibly recognized by BER. In addition, we investigated the consequences of the presence of βLdNs in a plasmid reporter system transcribed in human cells. Overall, βLdNs mostly behaved as non-instructive and non-repairable lesions but surprising exceptions were found for some DNA polymerases in vitro and for the fate of purine βLdNs in situ.

## 2. Results

### 2.1. dNMP Incorporation by DNA Polymerases Opposite βLdNs

**Figure 2:**
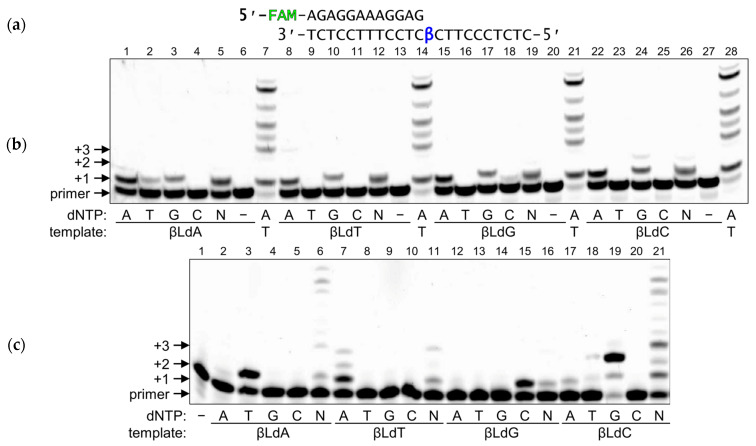
Primer extension by RBpol and Pol κ across βLdNs on a primer–template substrate. (**a**) Scheme of the substrate. FAM, fluorescent label; β, modified nucleotide. (**b**) Nucleotide incorporation by RBpol. (**c**) Nucleotide incorporation by Pol κ. +1 …+3, number of nucleotides added.

To address the mutagenic potential of βLdNs, we investigated the behavior of DNA polymerases belonging to different structural families on templates containing individually incorporated βLNs. As representative members of the polymerase families, here we studied the Klenow fragment of *E. coli* DNA polymerase I (3′→5′ exonuclease-deficient, KF, Family A), bacteriophage RB69 DNA polymerase (3′→5′ exonuclease-deficient, RBpol, Family B), human DNA polymerases β and λ (Pol β and Pol λ, Family X) and human DNA polymerase κ (Pol κ, Family Y). To investigate the ability of DNA polymerases to incorporate dNMPs opposite βLdNs we used a regular standing-start assay, where a modified nucleotide was located in the template immediately next to the 3′-end of the primer (Figure 2a).

Although KF is a highly efficient and processive DNA polymerase, it was completely blocked by any βLdN in the template (Supplementary Figure S1). Notably, this blockage was even stronger than that caused by truly non-coding abasic lesions and their adducts, which still can be bypassed by KF with low efficiency [26]. Increasing the enzyme concentrations did not improve KF’s ability to incorporate opposite βLdNs, in contrast to the undamaged substrate, which even showed some non-specific synthesis, as often observed in reactions with the excess of processive DNA polymerases (Supplementary Figure S1).

RBpol is a highly processive and accurate replicative phage DNA polymerase, structurally similar to eukaryotic replicative polymerases α, δ and ε. While its activity was also strongly blocked by βLdNs, increasing the enzyme concentration and the reaction time allowed us to achieve incorporation opposite βLdNs by RBpol (Figure 2a,b). Interestingly, RBpol preferred to incorporate dAMP and, to less extent, dGMP opposite all four βLdNs (Figure 2b, lanes 1, 3, 8, 10, 15, 17, 22, and 24). This preference is reminiscent of the “A-rule”, which some DNA polymerases follow when encountering non-coding lesions [27,28]. Moreover, trace amounts of correct (i.e., corresponding to base complementarity) nucleotide incorporation were detectable during the synthesis on βLdA- and βLdG-containing templates (dTMP and dCMP, respectively; Figure 2b, lanes 2 and 18). However, when all dNTPs were present in the reaction mixture, no extension after insertion at any βLdN was observed (Figure 2b, lanes 5, 12, 19, and 26). Interestingly, with both RBpol and other polymerases, we often observed that in the presence of all dNTPs even the first insertion was lower than with a single, preferentially used dNTP (e.g., compare lanes 15 and 19 or 22 and 26 in Figure 2b). The most likely reason is the nucleotide dilution effect. Unlike in the normal situation, when the complementary dNTP is bound with a strong preference over wrong ones, the *K*_d_s of dNTP binding opposite a lesion is higher and much less discriminate. We used either individual dNTPs at 500 µM, or a mixture of 125 µM for each dNTP, so less of any preferentially incorporated dNTP is available, and, additionally, poorly incorporated dNTPs compete with better-used ones for binding.

Translesion DNA polymerase Pol κ has the lowest intrinsic fidelity of all template-dependent DNA polymerases. Despite that, it predominantly incorporated correct dNMPs opposite βLdNs in the template (Figure 2c, lanes 3, 7, 15, and 19). Pol κ also efficiently continued the synthesis after the incorporation opposite to βLdA and βLdC in the presence of all four dNTPs (Figure 2c, lanes 6 and 21), consistent with its proposed major role as an “extender” polymerase during translesion synthesis [29].

**Figure 3:**
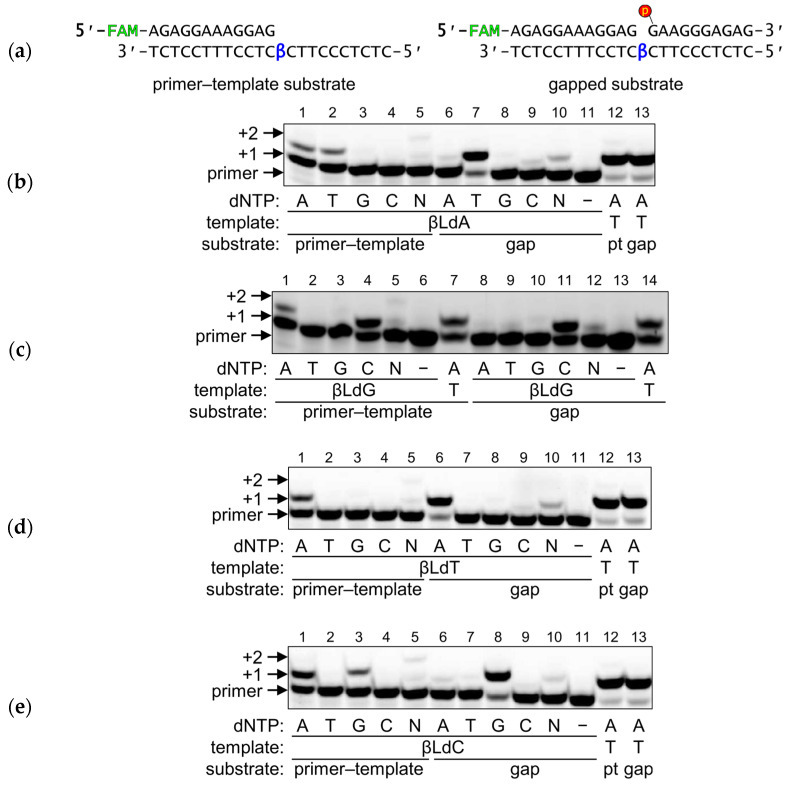
Primer extension by Pol β across βLdNs on primer–template(pt) and gapped (gap) substrates. (**a**) Scheme of the substrates. FAM, fluorescent label; β, modified nucleotide; p, 5′-terminal phosphate. (**b**–**e**) Insertion of dNMPs by Pol β opposite to βLdA (**b**), βLdG (**c**), βLdT (**d**) and βLdC (**e**). +1 …+2, number of nucleotides added.

In contrast to Family A and B enzymes, Family X DNA polymerases have relatively low processivity and lower fidelity. Surprisingly, Pol β demonstrated rather efficient synthesis on the βLdN-containing templates (Figure 3). The incorporation was lower than on undamaged substrates but comparable with—or even higher than—synthesis on non-coding templates (Supplementary Figure S2). Strikingly, on the primer–template substrates (Figure 3a), Pol β incorporated both a correct dNMP and dAMP, the latter again corresponding to the “A-rule”. Since Family X DNA polymerases prefer gapped substrates, as their main role is to fill small gaps during DNA repair, we next assessed the ability of Pol β to fill single-nucleotide gaps presenting βLdNs in the template (Figure 3a). The presence of the 5′-phosphorylated downstream strand not only increased the nucleotide incorporation efficiency but also improved the fidelity: Pol β readily extended the primer with the correct nucleotide while the incorporation of dAMP was markedly reduced (Figure 3b–e, compare lanes marked “primer–template” and “gap”). Thus, on its canonical substrate, Pol β was able to perform efficient and mostly accurate synthesis opposite βLdNs. In quite the opposite way, Pol λ, which is structurally similar to Pol β, was completely blocked by βLdNs on both primer–template and gapped substrates (Supplementary Figure S3).

### 2.2. Kinetic Basis for dNMP Incorporation Preference

As shown above, some DNA polymerases, particularly RBpol and Pol β, demonstrate a mixed type of incorporation with either “A-rule”-type or correct selection of the incoming nucleotide. To obtain better insight into the miscoding properties of βLdNs, we determined steady-state catalytic parameters of dNMP incorporation by RBpol and Pol β using the same substrates as in the primer extension experiments (Table 1). The results for RBpol were again consistent with the A-rule: dAMP was preferred over dGMP in all cases (as judged from the specificity constant, *k*_cat_/*K*_M_), and the template efficiency was in the order βLdA > βLdG > βLdC > βLdT for dAMP and βLdA > βLdC ≈ βLdG ≈ βLdT for dGMP incorporation. No apparent preference for the correct nucleotide selection (i.e., dAMP:βLdT or dGMP:βLdC) was found. However, under steady-state conditions we were unable to see significant incorporation of dCMP and dTMP opposite any βLdN. Moreover, even with dAMP and dGMP, enzyme saturation by the dNTP substrate could be achieved only for dAMP incorporation opposite βLdA and βLdG, indicating low affinity of dNTPs for the polymerase elongation complex encountering βLdNs.

When presented with a primer–template substrate, the kinetics of Pol β recapitulated its behavior in the qualitative experiments described above (Table 2). The insertion of dAMP was observed opposite all βLdNs in the order of preference βLdC > βLdT > βLdG ≥ βLdA. The correct pairs were formed with the efficiency dCMP:βLdG ≈ dAMP:βLdT > dGMP:βLdC > dTMP:βLdA, and, with the exception of the last one, the *k*_cat_/*K*_M_ values for all A-rule and correct primer extension events were within the same order of magnitude. In contrast, only the correct incorporation was evident with the gapped substrate (Table 3), in the order of preference dCMP:βLdG > dGMP:βLdC ≈ dTMP:βLdA > dAMP:βLdT. Interestingly, the reaction efficiency increased significantly for correct dCMP, dGMP and dTMP incorporation (13-, 15- and 113-fold, respectively) on the gapped substrate, while for dAMP incorporation the improvement was only 1.3-fold. No significant dAMP incorporation opposite to βLdA, βLdC, or βLdT was observed, indicating complete disappearance of the A-rule on the gapped substrate.

### 2.3. Enzyme-Dependent Recognition of βLdNs as Opposite Nucleotides by DNA Glycosylases

DNA glycosylases, the main enzymes that initiate the process of base excision repair, differ in their substrate specificity and reaction chemistry. Based on their mechanism, these are divided into monofunctional and bifunctional enzymes. Monofunctional glycosylases use a water molecule as a nucleophile that displaces the target nucleobase resulting in the formation of an apurinic/apyrimidinic (AP) site. In bifunctional glycosylases, the nucleophile is an enzyme’s amino group that forms a transient covalent intermediate (often referred in the literature as a Schiff base) with the C1′ atom of the target nucleotide. Subsequently, a series of electronic rearrangements leads to the elimination of the 3′-phosphate group (β-elimination). Notably, some DNA glycosylases, such as *E. coli* MutY and human MUTYH, recognize and remove not damaged nucleobases but normal ones that are improperly paired in DNA, while others (e.g., human TDG and MBD4) can excise both damaged and normal mispaired bases.

Using a collection of DNA glycosylases encompassing nearly all known substrate specificities, we have screened them for their ability to recognize and remove βLdNs from DNA. The panel of monofunctional glycosylases included Ung and MutY (the catalytic p25 domain) from *E. coli*, vaccinia virus D4 protein, and human MBD4 (catalytic domain), MPG, SMUG1, and UNG. The panel of bifunctional glycosylases comprised Fpg, Nei, and Nth from *E. coli* and human NEIL1, NEIL2, NEIL3, and OGG1 proteins. First, we used substrates in which βLdNs were placed opposite to any of the four canonical bases, as well as in single-stranded DNA. Despite observing some minor non-specific degradation in a few instances when using an excess of enzyme (20-fold) and a prolonged incubation time (1 h), we found that none of the DNA glycosylases tested were capable of efficiently cleaving any of the βLdN substrates. This was true even for MutY and MBD4, which, respectively, can remove unmodified A from A:G and A:8-oxoguanine mispairs and unmodified T from T:G mispairs. Based on these findings, we can conclude that DNA glycosylases are unable to cleave βLdNs regardless of the catalytic mechanism they employ.

At the next stage, we examined whether βLdNs, when paired with a damaged nucleotide specific for a DNA glycosylase, could impede its removal. Two monofunctional (MutY and MBD4) and two bifunctional DNA glycosylases (Fpg and OGG1) highly specific for the base opposite the target nucleotide were chosen for a more detailed investigation of their ability to process DNA in the presence of βLdNs.

**Figure 4:**
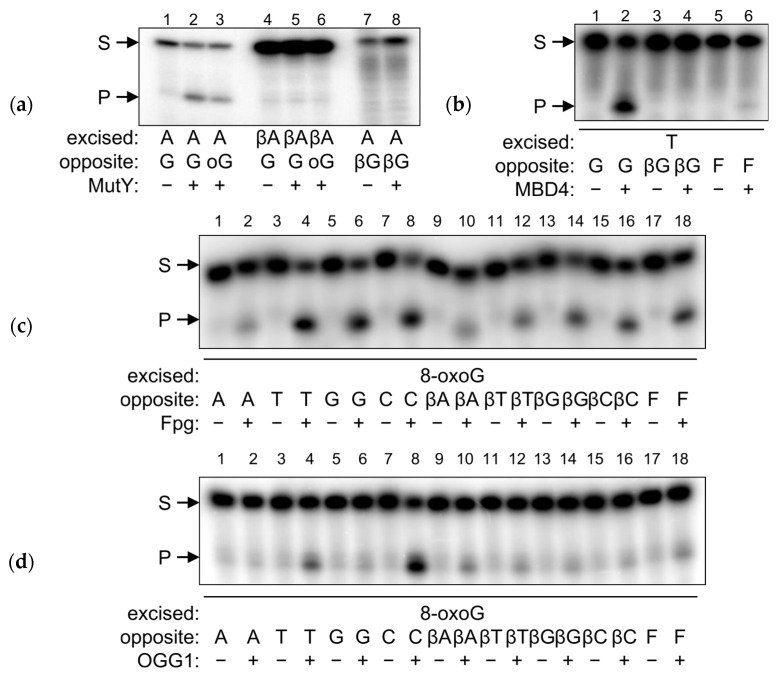
Cleavage of substrates containing βLdNs by DNA glycosylases MutY (**a**), MBD4 (**b**), Fpg (**c**) and OGG1 (**d**). S, oligonucleotide substrate; P, cleavage product. βA, βLdA; βC, βLdC; βG, βLdG; βT, βLdT.

For the MutY protein, which initiates the removal of A from A:8-oxoG and A:G mispairs [31], we investigated its ability to recognize and process βLdA opposite to G or 8-oxoG, as well as the normal A opposite to βLdG. As mentioned above, βLdA opposite to G was not subject to repair by MutY; neither was βLdA opposite to 8-oxoG (Figure 4a, lanes 1–6). Remarkably, when A was placed opposite to βLdG, this pair also was not processed by MutY (Figure 4a, lanes 7, 8). Similarly, no activity was observed for MBD4 when it was presented with the T:βLdG duplex, although it readily excised T from a T:G pair (Figure 4b).

The situation was different for human 8-oxoguanine DNA glycosylase (OGG1) and *E. coli* formamidopyrimidine–DNA glycosylase (Fpg). Despite sharing neither homology nor three-dimensional fold, these enzymes have essentially identical substrate specificities. Normally, both Fpg and OGG1 have a marked preference for 8-oxoG:C pairs and strongly discriminate against 8-oxoG:A [32]. This specificity prevents the accumulation of G→T transversions caused by incorrect dAMP incorporation opposite 8-oxoG by DNA polymerases. The presence of any βLdN opposite the lesion reduced the efficiency of 8-oxoG removal by both proteins but did not completely eliminate it (Figure 4c,d). The effect was more pronounced for OGG1, for which the residual activity was similar for all βLdNs opposite to 8-oxoG. Notably, both Fpg and OGG1 processed 8-oxoG:βLdN pairs with the efficiency similar to 8-oxoG paired with (3-hydroxytetrahydrofuran-2-yl)methyl phosphate (F), a stable AP site analog that has no base-pairing capability (Figure 4c,d, lanes 17 and 18).

For quantitative characterization of the observed activity, we also compared the kinetic parameters of Fpg and OGG1 on duplex substrates containing C, βLdC or F opposite to 8-oxoG. Indeed, for Fpg, both *K*_M_ and *k*_cat_ with 8-oxoG:βLdC and 8-oxoG:F substrates were the same within the error margin (Table 4). At the same time, *K*_M_ for these substrates was 3.9–4.5-fold higher and *k*_cat_ 2.5-fold lower than the corresponding values for 8-oxoG:C, amounting to about an order of magnitude preference for the canonical substrate (Table 4). OGG1, due to the slow product release, is usually kinetically characterized in terms of its glycosylase reaction rate constant (*k*_2_) determined in single-turnover experiments [33,34]. The observed *k*_2_ values for 8-oxoG:βLdC and 8-oxoG:F substrates were again similar and were 40–50-fold lower than for the canonical substrate. Thus, for both Fpg and OGG1, βLdC behaved as a non-cognate opposite nucleotide.

### 2.4. βLdNs Are Resistant to AP Endonucleases

**Figure 5:**
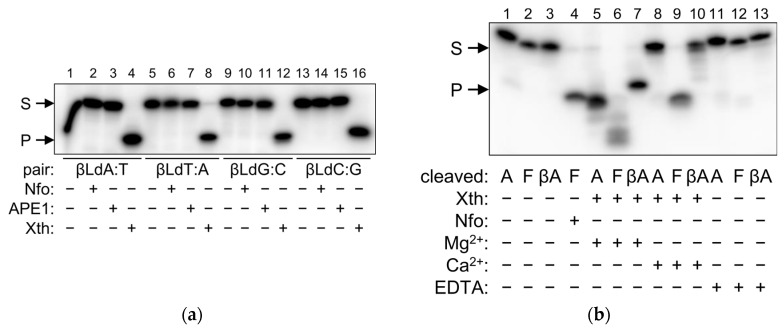
Cleavage of substrates containing βLdNs by AP endonucleases. (**a**) Cleavage of βLdA:T, βLdT:A, βLdG:C and βLdC:G substrates by human APE1 and *E. coli* Nfo and Xth. (**b**) Cleavage of βLdA:T by Xth under different conditions. S, substrate, P, cleavage product.

As shown above, in some respects βLdNs may behave as abasic DNA units when interacting with DNA polymerases and DNA glycosylases. Thus, we inquired whether AP endonucleases, the enzymes naturally involved in AP site repair, can recognize and cleave βLdNs in double-stranded DNA. We have tested *E. coli* Xth and Nfo, two AP endonucleases belonging to different structural superfamilies (exonuclease–endonuclease–phosphatase and triosephosphate isomerase barrel, respectively), and human APE1, an Xth homolog. APE1 and Nfo were not able to process any βLdN, whereas a cleavage band was observed with Xth (Figure 5a, lanes 4, 8, 12, and 16). However, this was likely a consequence of the exonuclease activity of Xth degrading the duplex in the 3′→5′ direction and stalling at the βLdN position. When we replaced Mg^2+^ with Ca^2+^ in the reaction buffer (which is much less supportive for the exonuclease activity) and lowered the enzyme concentration and incubation time, Xth still robustly processed F-containing substrates but failed to cleave at βLdNs (Figure 5b, compare lanes 5–7 and 8–10). It can also be noted that Mg^2+^-dependent substrate degradation by Xth produced a band of higher mobility than the product of F cleavage by Xth or Nfo (Figure 5b, compare lanes 4, 5 and 7; both Xth and Nfo cleave 5′ to F), suggesting that Xth does not hydrolyze the phosphodiester bond 5′ of the βLdN. We conclude that βLdNs in double-stranded DNA cannot be processed by AP endonucleases.

### 2.5. Repair of βLdNs in Human Cells

**Figure 6:**
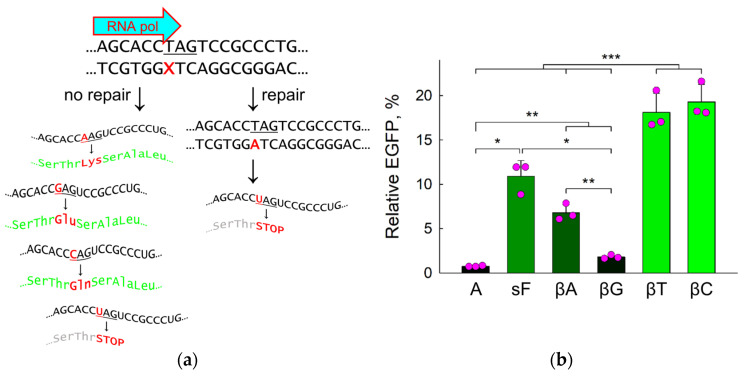
Transcriptional mutagenesis and repair induced by βLdNs in HeLa cells. (**a**) Scheme of the processes induced by a lesion in the *eGFP* reporter gene. (**b**) Relative EGFP expression normalized for the fluorescence of the control G-construct (*n* = 3, mean ± SD shown). Differences between constructs: *p* < 0.05 (*); *p* < 0.01 (**); *p* < 0.005 (***); two-tailed Student’s *t*-test with Bonferroni correction applied. The brightness of the bars is proportional to the EGFP expression level.

Finally, to address the fate of βLdN modifications in living cells, we used the previously described system of reporter plasmids containing non-fluorescent variants of EGFP [35,36]. A c.613C>T mutation in the eGFP sequence introduces a stop codon leading to a truncated protein (p.Q205X). When a miscoding or non-instructive nucleotide is placed at this position in the transcribed strand, RNA polymerase II may incorporate a ribonucleotide other than UMP opposite the lesion, with all three arising protein variants (205Q, 205E and 205K) showing fluorescence (Figure 6a). If the lesion is repaired in the cell, the non-fluorescent truncated EGFP variant is restored. The pZAJ vector carrying the lesion does not replicate in the cell line used (HeLa), thus eliminating the possibility of fluorescence arising due to replication-associated mutagenesis at the modification site [37].

When βLdNs or normal nucleotides were incorporated in position 613 of the eGFP transcribed strand, the fluorescence of the mutant construct with A was, as expected, <1% of the fully active wild-type G-construct (Figure 6b). If βLdA was placed at this position instead, we observed a severalfold increase in fluorescence (to ~7%), indicative of the error-prone bypass (Figure 6b). This level was slightly below the fluorescence of F modified with a 5′-phosphotioate linkage (sF), a model abasic lesion resistant to APE1 but subject to less efficient removal by some back-up pathways, possibly nucleotide excision repair (NER) [35,38]. Even larger fluorescent cell populations (~20% of the G-construct) were produced by βLdC and βLdT, suggesting either slower repair of these nucleotides or their higher miscoding potential (Figure 6b). Quite surprisingly, when βLdG was present in eGFP position 613, the fluorescence increased only 2.4-fold compared to the A negative control, which was significantly less than with the other βLdNs and sF (Figure 6b). Apparently, either βLdG is nearly fully repaired in the cell or RNA polymerase II incorporates preferentially UMP opposite to this modification.

## 3. Discussion

Nucleic acid chains made of β-L-deoxynucleotides, the mirror images of their naturally occurring D-enantiomer counterparts, are promising tools for many applications due to their predictable base-pairing ability and excellent stability against nucleases [6]. Oligonucleotides fully consisting of βLdNs have the same thermodynamic properties as D-oligonucleotides and form duplexes of the opposite chirality to the identical D-DNA sequences [39,40,41,42]. Nevertheless, they cannot form Watson–Crick pairs with their complementary D-DNA strands [43].

While the properties of fully L-DNA have been subject to many studies, much less is known about DNA with embedded single βLdNs. As the structure of such duplexes is concerned, NMR studies [19,21,22] show that individual L-nucleotides maintain Watson–Crick-type hydrogen bonding, indicating substantial conformational pliability. This situation is somewhat reminiscent of the properties of α-anomeric dA, which, when occurring as a single residue within DNA, is found within the double helix forming reverse Watson–Crick pairs with dT with only slight to moderate backbone deformation [44,45]. Nevertheless, almost nothing is known about the biochemistry of βLdNs within the otherwise normal DNA; in particular, their ability to serve as templates for DNA polymerases or their possible recognition as lesions by DNA repair enzymes. Reports on the template properties of βLdN are contradictory: it has been stated that βLdA is not bypassed by KF, *Taq* DNA polymerase and avian myeloblastosis virus reverse transcriptase [46] and that several thermophilic DNA polymerases used in PCR are temporarily stalled at βLdT but eventually bypass up to three consecutive L-chiral units in an error-free manner [47]. Obviously, differences in the enzyme and nucleobase nature and in the reaction conditions could be responsible for these discrepancies, so a systematic comparison is required to assess the capability of DNA polymerases to incorporate dNMPs opposite to βLdNs.

DNA polymerases encountering modified deoxynucleotides can perceive them as instructive (preferentially directing incorporation of a single dNMP, regardless of its complementarity to the original template nucleotide), non-instructive (incorporating a dNMP in a template-independent manner) or blocking (unable to bypass), with non-instructive modifications usually being partially blocking. The best known non-instructive lesions are AP sites, which, by their chemical nature, cannot form hydrogen bonds with an incoming dNTP. A well-established property of AP sites, termed the “A-rule”, is the tendency of many (but not all) DNA polymerases to incorporate dAMP opposite abasic lesions [27,28]. In different families of DNA polymerases, this preference arises from the use of an enzyme’s amino acid as a substitute for base pairing or from better stacking of adenine with the primer–template junction as compared with other bases [48,49], or may be guided by fine changes in the enzyme’s conformational dynamics [50]. In our experiments, βLdNs very unexpectedly behaved as instructive, “A-rule” non-instructive or blocking depending on the type of DNA polymerase and even on the structure of the substrate. To our knowledge, this is the first example of modified nucleotides possessing such features.

KF was completely blocked by βLdNs of any type, consistent with the βLdA data reported earlier [46]. Among all polymerases studied here, KF’s active site is the most restrained around the template nucleotide due to a sharp kink in the template strand immediately next to it [51]. The possibility of conformational adjustment of the phosphodiester backbone to position a βLdN residue appears to be severely limited in this case. On the opposite end, Pol κ, which has the most relaxed active site, demonstrated almost exclusively the incorporation of complementary dNMPs opposite to βLdNs. The template strand bound to Pol κ is open into a wide and accessible groove, allowing the enzyme to efficiently accommodate even strongly distorting bulky lesions such as cyclobutane pyrimidine dimers and cisplatin cross-links [52,53]. Thus, binding of a correct dNTP is likely sufficient to adjust the backbone conformation and stabilize a βLdN in a canonical base pair in the active site.

RBpol was strongly blocked by βLdNs but, at increased concentrations, was able to incorporate mostly dAMP and dGMP regardless of the template base. In our earlier experiments with substrates of the same sequence, RBpol also inserted dAMP, dGMP and, to a much lesser extent, dTMP opposite the natural AP site [30]. Our results are also concordant with an extensive set of pre-steady-state kinetic data on RBpol bypassing an F abasic site with different base pairs at the primer–template junction, where the enzyme showed the general preference A > G >> T > C [54]. As the preference of RBpol for incorporation opposite the truly non-instructive abasic lesions and opposite βLdNs is the same, we suppose that βLdNs in the active site adopt a conformation that does not present the Watson–Crick face towards the incoming dNTP, and the incorporation is guided predominantly by A or G stacking against the base pair at the primer–template junction, as with F [48,54]. Notably, βLdN-containing templates were used by the enzyme with a 4.1–5.2-fold higher *K*_M_ and a 11–23-fold lower *k*_cat_ in comparison with the AP substrate (Table 1), resulting in an overall 47–290-fold lower efficiency (*k*_cat_/*K*_M_) and indicating significant steric hurdles in forming the catalytically competent ternary complex. A dNTP-stabilized template misalignment, another frequently observed mechanism of misincorporation opposite DNA lesions, is less likely to operate in this situation, since our substrates have C at +1 position and would guide preferential incorporation of dGMP rather than dAMP.

The case of Pol β was the most intriguing for it partly adhered to the “A-rule” on the primer–template substrate but completely lost this propensity on the gapped substrate, incorporating only complementary nucleotides instead. Of all DNA polymerases, Pol β is noted for its strong preference for the presence of a 5′-phosphorylated downstream strand, which is contacted by Pol β’s N-terminal domain to load the enzyme on DNA [55]. In fact, natural AP site in the absence of a downstream strand blocks the elongation completely [30] while F can be partially bypassed in a surrounding sequence-dependent manner at large enzyme excess [56]. Bound to a gapped substrate, Pol β can induce DNA backbone adjustment to accommodate distorting DNA lesions such as spiroiminodihydantoin [57], cisplatin-G^G cross-links [58] or α-anomer of 2,6-diamino-4-hydroxy-5-formamidopyrimidine [59] in a conformation permissive for slow yet still template-guided (as opposed to non-instructive) bypass. Compared with the natural AP site in exactly the same sequence context [30], gapped βLdN-containing substrates are utilized with a similar or even better efficiency due to their higher *k*_cat_ (Table 3). It should be noted that the steady-state reaction rate for nucleotide misincorporation or incorporation on damaged templates by Pol β is limited by the chemical step rather than by product release as in the correct incorporation [60]. Thus, it is quite feasible that, as with Pol κ, the incoming complementary dNTP stabilizes the “correct” base pair enough to populate the catalytically competent conformation of the active site at least partially.

As DNA repair is concerned, DNA glycosylases and AP endonucleases did not process βLdNs. DNA glycosylases typically remove damaged bases but some of them, such as bacterial MutY, its eukaryotic homolog MUTYH, and eukaryotic MBD4, can excise normal bases from mismatches. However, neither *E. coli* MutY nor human MBD4 cleaved duplexes with βLdA:G/8-oxoG, A:βLdG, βLdT:G and T:βLdG, which correspond to their respective natural substrates A:G/8-oxoG and T:G. Target nucleotide recognition by most DNA glycosylases requires its extrusion from the double helix, insertion into the enzyme’s active site, and a nucleophilic attack at C1′ [61,62,63]. Moreover, to distinguish between normal base pairs and mismatches, MutY and MBD4 also extensively probe for the nature of the opposite base. The whole process is effected through a highly co-ordinated series of conformational changes in the enzyme–substrate complex and is apparently impossible with the alternative nucleotide configuration. Fpg and OGG1 seem to be an exception: while being less active than on their natural substrate (8-oxoG:C), they still processed 8-oxoG paired with βLdNs, and did so with the efficiency comparable with 8-oxoG placed opposite an F abasic site. Both Fpg and OGG1 have been suggested to initiate base extrusion through active local destabilization of the Watson–Crick-type 8-oxoG:C pair [64,65,66], so perhaps pre-existing destabilization by a βLdN or an abasic site promotes the reaction. In fact, Fpg, while usually regarded as 8-oxoG:C-specific, also efficiently removes 8-oxoG mispaired with G or T [67], which was also observed in our experiments (Figure 4c). So, it seems that βLdNs behave as strictly non-instructive with DNA glycosylases, and the ability of these enzymes to process base pairs with such modifications strongly depends on the mechanism of target recognition. Their non-instructive nature notwithstanding, the repair of βLdNs should be quite different from the repair of AP sites, since the former are resistant to AP endonucleases, which efficiently cleave DNA at abasic nucleotides of different chemical nature [68,69].

Even if not through BER, can βLdNs undergo repair in the cell at all? Our fluorescent reporter data reveal an unexpected difference between pyrimidine- and purine-bearing βLdNs in this respect. Plasmids containing βLdC and βLdT produced a significantly larger population of cells expressing functional eGFP than βLdA- and especially βLdG-carrying constructs. In the reporter system used here (Figure 6a), non-fluorescent eGFP molecules are produced either when UMP is incorporated opposite a template βLdN by RNA polymerase II, or when a βLdN is repaired, which yields A in the template strand since the modification is placed opposite to T. We did not explicitly address the efficiency and specificity of RNA polymerase II bypass of βLdNs; however, it has been shown that RNA and DNA polymerases generally follow the same misincorporation pattern on damaged templates of different natures [70,71,72,73,74,75]. From the behavior of DNA polymerases studied here, we do not expect significant preference for non-instructive UMP incorporation opposite βLdNs by RNA polymerases, and repair remains the most likely reason for low fluorescence. The fluorescence of βLdG-transfected cells was significantly lower than that of the cells transfected by sF, a known modification resistant to BER but slowly repaired through an unidentified pathway, possibly NER [35,38]. We suggest that at least βLdG is subject to repair in human cells, although NER is unlikely to play a role here since the human NER system does not recognize single βLdG residues [76]. The same could be true for βLdA: whereas in this case the difference from sF did not reach statistical significance, the fluorescence of this cell population was significantly below the cells transfected with βLdC and βLdT plasmids. However, with βLdA, we cannot exclude that complementary UMP incorporation also contributes to the overall fluorescence decrease. Mismatch repair could also be involved if βLdNs paired with normal nucleotides are recognized by the MSH2/MSH6 complex, which has the ability to bind many kinds of DNA lesions in addition to mismatches [77]. The pyrimidine βL modifications, on the other hand, are repaired inefficiently, if at all. The mechanisms of βLdG repair and the behavior of RNA polymerases encountering βLdNs remain a subject of further study.

Overall, our data present βLdNs as a unique kind of DNA modification when DNA repair and replication is concerned. Despite their apparent ability to form hydrogen-bonded pairs in duplex DNA, βLdNs act as non-instructive lesions for DNA polymerases and opposite base-specific DNA glycosylases, or have context-dependent dual non-instructive/instructive properties in special cases such as DNA synthesis by Pol β or Pol κ. Thus, βLdNs may serve as a useful model in studies of cellular response to presumably orthogonal non-natural DNA modifications.

## 4. Materials and Methods

### 4.1. Enzymes, Oligonucleotides, Plasmids and Cells

The following enzymes were overexpressed and purified essentially as described earlier: *E. coli* Fpg [78], MutY (the catalytic p25 fragment) [79], Nei [80], Nth [81], Nfo [82], human OGG1 [83], MBD4 (the catalytic fragment) [62], NEIL1 [84], NEIL2 [85], vaccinia virus D4 protein [86], exonuclease-deficient KF [87], exonuclease-deficient RBpol [88], human Pol β [89], the catalytic fragment (residues 242–575) of human Pol λ [90] and the catalytic fragment (residues 1–560) of human Pol κ [91]. Human MPG, NEIL3 and SMUG1 proteins were kindly provided by Dr. Murat Saparbaev (Institut Gustave Roussy, France). *E. coli* Xth and Ung were from SibEnzyme (Novosibirsk, Russia). Oligonucleotides (Supplementary Table S1) were synthesized in-house from commercially available phosphoramidites (Glen Research, Sterling, VA, USA). If necessary, the oligonucleotides were end-labeled with γ[^32^P]ATP (ICBFM Laboratory of Biotechnology, Novosibirsk, Russia) and phage T4 polynucleotide kinase (Biosan, Novosibirsk, Russia) and desalted on an Isolute C18 sorbent (Biotage, Uppsala, Sweden). Plasmids pZAJ_Q205* and pZAJ_5c were a kind gift of Dr. Andriy Khobta (Friedrich Schiller University Jena, Germany). HeLa cells were from the laboratory stock and were tested for the absence of mycoplasma contamination by PCR.

### 4.2. DNA Polymerases Standing-Start Assay

The reaction mixture (10 µL) included 100 nM fluorescein-labeled primer, 200 nM βLdN-containing template, 200 nM downstream strand (when necessary), 500 µM dNTP (either individual or an equimolar mixture of each) and 5 nM Pol β or Pol κ or 20 nM RBpol or KF or Pol λ in 50 mM Tris–HCl (pH 7.5, or pH 8.5 for Pol λ), MgCl_2_ (10 mM for Pol β, 2.5 mM for Pol λ, 5 mM for other polymerases) and 1 mM DTT. The reaction was allowed to proceed for 10 min at 25 °C and stopped by adding an equal volume of 10 mM EDTA in formamide and heating at 95 °C for 2 min. The reaction products were resolved by electrophoresis in 20% polyacrylamide gel/7.2 M urea and visualized using a Typhoon FLA 9500 phosphorimager (GE Healthcare, Chicago, IL, USA).

### 4.3. DNA Polymerases Steady-State Kinetics

The reaction mixtures were assembled, run and processed as above but with the concentrations of dNTP varying from 5 µM to 1000 µM. The DNA polymerase concentration and reaction time for each enzyme–substrate pair was optimized to yield less than 20% primer extension (Supplementary Table S2). The products were quantified using Quantity One v4.6.3 software (Bio-Rad Laboratories, Hercules, CA, USA). The data were fitted to the Michaelis–Menten equation using SigmaPlot v11.0 (Systat Software, Chicago, IL, USA). All reported constants are derived from three to four independent experiments.

### 4.4. DNA Glycosylase Assay

The reaction mixture (10 µL) included 50 mM Tris–HCl (pH 7.5), 100 mM NaCl, 1 mM EDTA, 1 mM DTT, 50 nM double-stranded substrate (Supplementary Table S1) and 500 nM MutY, or 200 nM MBD4, or 50 nM Fpg, or 40 nM OGG1. The mixtures were incubated at 37 °C for 1 h (MutY) or 15 min (all other glycosylases). For monofunctional DNA glycosylases and OGG1, the reaction was terminated by adding NaOH to 0.1 M, heating for 5 min at 95 °C and neutralizing with an equimolar amount of HCl. After that, or immediately after the reaction for bifunctional glycosylases, 5 µL of the gel loading dye (20 mM EDTA, 0.1% xylene cyanol, 0.1% bromophenol blue in 80% formamide) followed by heating at 95 °C for 3 min. The reaction products were resolved, visualized and quantified as described above.

### 4.5. Steady-State Fpg Kinetics

The reaction mixture (10 µL) included the glycosylase reaction buffer (as above), 3–100 nM oxoG-containing ^32^P-labeled duplex and 1 nM Fpg (active enzyme). The mixtures were incubated for 10 min, processed and analyzed as above. The product amount did not exceed 15% of the total substrate. *K*_M_ and *k*_cat_ were determined by data fitting to the hyperbolic equation *v* = *k*_cat_[E]_0_[S]_0_/(*K*_M_ + [S]_0_) using SigmaPlot v11.0. All quantitative parameters and data reported in figures and tables were determined from 3–4 independent experiments.

### 4.6. Singe-Turnover OGG1 Kinetics

The reaction mixture (50 µL) included the glycosylase reaction buffer (as above), 100 nM oxoG-containing ^32^P-labeled duplex and 2 µM OGG1 (total protein). The reactions were allowed to proceed for 0–60 min at 37 °C; 5-μL aliquots were withdrawn at 20 s, 40 s, 1, 3, 5, 10, 20, 40, and 60 min and quenched by mixing with 1 µL of 1 M NaOH followed by heating at 95 °C for 3 min. The samples were neutralized with an equimolar amount of HCl, mixed with gel loading dye, processed and analyzed as above. The apparent base excision rate constant, *k*_2app_, was determined by fitting the data to the equation $\left[P\right]=A\left(1-e^{-k_{2app}t}\right)$ where [P] is the product concentration, A is the maximal cleavage and *t* is time using SigmaPlot v11.0.

### 4.7. AP Endonuclease Assay

The reaction mixture (10 µL) included 20 mM HEPES–NaOH (pH 7.5), 50 mM KCl, 1 mM DTT, 5 mM MgCl_2_ or 5 mM CaCl_2_ or 1 mM EDTA, 50 nM double-stranded substrate (Supplementary Table S1). In the initial screening of all AP endonucleases, APE1 in the reaction mixture was taken at 1000 nM, Nfo, at 400 nM, and Xth, at 200 nM, and the reactions were incubated at 37 °C for 1 h. In the follow-up experiments with Xth, 10 nM enzyme was used, and the reaction time was 10 min. The reactions were terminated and processed as above.

### 4.8. Transcription Mutagenesis Experiments

Plasmids pZAJ_Q205* carrying βLdN modifications or canonical nucleotides in the *eGFP* reporter gene between two Nb.Bpu10I nickase recognition sites were generated as previously described [38,92]. The pZAJ_5c plasmid with the original EGFP sequence [37] was used for fluorescence data normalization. HeLa cells (10^5^ per well) were seeded in a 12-well plate. After 24 h, 100 ng of the construct with a βLdN modification together with 300 ng of pDsRed-Monomer-N1 (Takara Bio, Kusatsu, Japan) were transfected using Effectene transfection reagent (Qiagen, Venlo, The Netherlands). Twenty-four hours post-transfection, cells were collected, fixed in 1% formaldehyde solution, and analyzed on a Novocyte 3000 flow cytometer (Agilent Technologies, Santa Clara, CA, USA). The median green fluorescence of EGFP^+^ DsRed^+^ cells transfected with βLdN-carrying pZAJ_Q205* was normalized for the median green fluorescence of EGFP^+^ DsRed^+^ pZAJ-5c-transfected cells [35,93,94].

### 4.9. Structure Modeling

The structure of trinucleotide 5′-T[βLdT]C-3′/5′-GAA-3′ shown in Figure 1b was obtained from the structure of trinucleotide 5′-TTC-3′/5′-GAA-3′, a part of the normal Dickerson dodecamer (PDB ID 355D [12]) using ChemSketch v11.0 (Advanced Chemistry Development, Toronto, ON, Canada). Explicit hydrogens were added and the .pdb co-ordinates of 5′-TTC-3′/5′-GAA-3′ were converted to MDL Molfile V2000 format using PyMol v0.99. The chirality of the central dT was manually inverted in ChemSkecth, hydrogen bonds were added, and the whole structure was subjected to optimization of bond lengths, planar and dihedral angles, and non-bonded interactions using the 3D optimization module of ChemSketch based on the CHARMM force field [95].

## Figures and Tables

**Table 1:** Steady-state kinetic parameters for dNMP incorporation opposite βLdNs by RBpol *^a^*.

Template	dNTP	*K*_M_, µM	*k*_cat_, s^−1^, ×10^4^	*k*_cat_/*K*_M_, µM^−1^ × s^−1^, ×10^6^
βLdA	dATP	450 ± 130	24 ± 3	5.3 ± 1.7
βLdT	dATP	n/s *^b^*	n/s	0.85 ± 0.04
βLdG	dATP	570 ± 180	12 ± 2	2.1 ± 0.8
βLdC	dATP	n/s	n/s	1.6 ± 0.1
βLdC	dGTP	n/s	n/s	0.78 ± 0.07
βLdT	dGTP	n/s	n/s	0.73 ± 0.03
βLdG	dGTP	n/s	n/s	0.77 ± 0.04
βLdA	dGTP	n/s	n/s	1.6 ± 0.1
AP *^c^*	dATP	110 ± 30	270 ± 30	250 ± 70

*^a^* Mean ± s.e.m from three to four independent experiments. Formation of other pairs was negligible under the conditions used. *^b^* n/s, not saturated; saturation of the enzyme was not achieved in the used dNTP range, *k*_sp_ was determined from the linear slope of the *v*_0_ vs. [S]_0 (dNTP)_ dependence. *^c^* Data from [30].

**Table 2:** Steady-state kinetic parameters for dNMP incorporation opposite βLdNs by Pol β on a primer–template substrate *^a^*.

Template	dNTP	*K*_M_, µM	*k*_cat_, s^−1^, ×10^4^	*k*_cat_/*K*_M_, µM^−1^ × s^−1^, ×10^5^
βLdA	dATP	64 ± 16	32 ± 2	5.0 ± 1.3
βLdT	dATP	53 ±18	100 ± 10	19 ± 7
βLdG	dATP	57 ± 9	38 ± 1	6.7 ± 1.1
βLdC	dATP	46 ± 11	210 ± 10	46 ± 11
βLdA	dTTP	n/s *^b^*	n/s	0.54 ± 0.02
βLdG	dCTP	120 ± 20	270 ± 20	23 ± 4
βLdC	dGTP	200 ± 50	98 ± 10	4.9 ± 1.3
AP *^c^*	dATP	no significant incorporation		

*^a^* Mean ± s.e.m from three to four independent experiments. Formation of other pairs was negligible under the conditions used. *^b^* n/s, not saturated; saturation of the enzyme was not achieved in the used dNTP range, *k*_sp_ was determined from the linear slope of the *v*_0_ vs. [S]_0 (dNTP)_ dependence. *^c^* Data from [30].

**Table 3:** Steady-state kinetic parameters for dNMP incorporation opposite βLdNs by Pol β on a gapped substrate *^a^*.

Template	dNTP	*K*_M_, µM	*k*_cat_, s^−1^, ×10^4^	*k*_cat_/*K*_M_, µM^−1^ × s^−1^, ×10^4^
βLdT	dATP	100 ± 20	240 ± 10	2.4 ± 0.5
βLdA	dTTP	67 ± 12	410 ± 20	6.1 ± 1.1
βLdG	dCTP	17 ± 3	510 ± 20	30 ± 5
βLdC	dGTP	37 ± 9	210 ± 20	7.3 ± 1.9
AP *^b^*	dATP	2.9 ± 1.5	4.4 ± 0.2	1.5 ± 0.8

*^a^* Mean ± s.e.m from three to four independent experiments. Formation of other pairs was negligible under the conditions used. *^b^* Data from [30].

**Table 4:** Kinetic parameters for cleavage of 8-oxoG-containing substrates by Fpg and OGG1 *^a^*.

Substrate	Fpg			OGG1
	*K*_M_, nM	*k*_cat_, min^−1^	*k*_cat_/*K*_M_, nM^−1^ × min^−1^, ×10^3^	*k*_2_, min^−1^
8-oxoG:C	9.3 ± 1.3	0.34 ± 0.01	37 ± 5	1.8 ± 0.2
8-oxoG:βLdC	42 ± 11	0.14 ± 0.02	3.3 ± 1.0	0.047 ± 0.006
8-oxoG:F	36 ± 18	0.13 ± 0.03	3.6 ± 2.0	0.038 ± 0.002

*^a^* Mean ± s.e.m from three to four independent experiments.

## Data Availability

All data are reported in the paper and the Supplementary Materials.

## Supplementary Materials

The following supporting information can be downloaded at: https://www.mdpi.com/article/10.3390/ijms25116006/s1.
