# Supplementary material for: Probing the Conformational Restraints of DNA Damage Recognition with β-L-Nucleotides
Source: Int J Mol Sci. 2024 May 30;25(11):6006. doi: 10.3390/ijms25116006 (PMC11172447; doi:10.3390/ijms25116006)
Supplement: Supplementary file 1 [file ijms-25-06006-s001.zip › ijms-3005949-supplementary.pdf]

SUPPLEMENTARY MATERIAL FOR

Probing the conformational restraints of DNA damage recognition with β-L-nucleotides

Anna V. Yudkina [1,2], Daria V. Kim [1], Timofey D. Zharkov [1], Dmitry O. Zharkov [1,2,*], and Anton V. Endutkin [1,*]

[1] Siberian Branch of the Russian Academy of Sciences Institute of Chemical Biology and Fundamental Medicine, 8 Lavrentieva Ave., 630090 Novosibirsk, Russia; ayudkina@niboch.nsc.ru (A.V.Y.); dkim@niboch.nsc.ru (D.V.K.); timazharkov74@gmail.com (T.D.Z.); aend@niboch.nsc.ru (A.V.E.)

[2] Department of Natural Sciences, Novosibirsk State University, 2 Pirogova St., 630090 Novosibirsk, Russia; dzharkov@niboch.nsc.ru (D.O.Z.)

* Correspondence: dzharkov@niboch.nsc.ru (D.O.Z.); aend@niboch.nsc.ru (A.V.E.)

This file contains:

- Supplementary Tables S1–S2
- Supplementary Figures S1–S3
- Supplementary References

Supplementary Table S1. Structure of DNA substrates and oligonucleotides used in this work.

**Base excision repair experiments: General duplex structure [1,2]**		
`5′-AGAGGAAAGGAGXGAAGGGAGAG-3′`<br>`3′-TCTCCTTTCCTCYCTTCCCTCTC-5′`	X = A, C, G, or T<br>Y = *βLdA, *βLdC, *βLdG, *βLdT, *A, or *F	
**Base excision repair experiments: Opposite base specificity**		
Enzyme	***X*** **=**	***Y*** **=**
MutY	G	*A
	8-oxoG	*A
	*A	βLdG
	G	*βLdA
	8-oxoG	*βLdA
MBD4	*T	G
	*T	βLdG
	*T	F
Fpg, OGG1	*8-oxoG	A
	*8-oxoG	C
	*8-oxoG	G
	*8-oxoG	T
	*8-oxoG	βLdA
	*8-oxoG	βLdC
	*8-oxoG	βLdG
	*8-oxoG	βLdT
	*8-oxoG	F
**DNA polymerase experiments [3,4]**		
`5′-[Fluo]AGAGGAAAGGAG`<br>`3′-TCTCCTTTCCTCXCTTCCCTCTC-5′`	primer–template substrate<br>X = T, βLdA, βLdC, βLdG, βLdT, or F	
`p`<br>`5′-[Fluo]AGAGGAAAGGAG GAAGGGAGAG-3′`<br>`3′-TCTCCTTTCCTCXCTTCCCTCTC-5′`	gapped substrate<br>X = T, βLdA, βLdC, βLdG, or βLdT	
**Transcription mutagenesis experiments**		
`5′-GCACCYAGTCCGCCCTGA-3′`	complementary oligonucleotide used to make a gapped plasmid [5]<br>Y = C or T	
`5′-TCAGGGCGGACTXGGTGC-3′`	oligonucleotide ligated into gap<br>X = G, A, sF, βLdA, βLdC, βLdG, or βLdT	

[1] Asterisk marks the ^{32}P-labeled strand.

[2] In the experiments with single-stranded substrates, only strand Y was used.

[3] [Fluo], 5(6)-carboxyfluorescein.

[4] The 5′-terminal G of the downstream strand in the gapped substrate was phosphorylated during the synthesis.

[5] The workflow of the reporter plasmid constriction is described in detail in {Lühnsdorf, 2012 #14387}{Kim, 2024 #19514}.

Supplementary Table S2. Optimized reaction conditions for steady-state DNA polymerase kinetics

Enzyme	DNA	dNTP	System	Enzyme, nM	Reaction time, min
Pol β	βLdA	dATP	primer–template	10	10
Pol β	βLdT	dATP	primer–template	3	10
Pol β	βLdG	dATP	primer–template	10	10
Pol β	βLdC	dATP	primer–template	1	10
Pol β	βLdA	dTTP	primer–template	10	10
Pol β	βLdG	dCTP	primer–template	2	10
Pol β	βLdC	dGTP	primer–template	5	10
Pol β	βLdA	dTTP	gap	1	10
Pol β	βLdT	dATP	gap	2	10
Pol β	βLdG	dCTP	gap	1	10
Pol β	βLdC	dGTP	gap	1	10
RBpol	βLdT	dATP	primer–template	20	20
RBpol	βLdC	dGTP	primer–template	20	20
RBpol	βLdA	dATP	primer–template	10	20
RBpol	βLdT	dGTP	primer–template	20	20
RBpol	βLdG	dGTP	primer–template	20	20
RBpol	βLdC	dATP	primer–template	10	20
RBpol	βLdA	dGTP	primer–template	10	20
RBpol	βLdG	dATP	primer–template	20	20

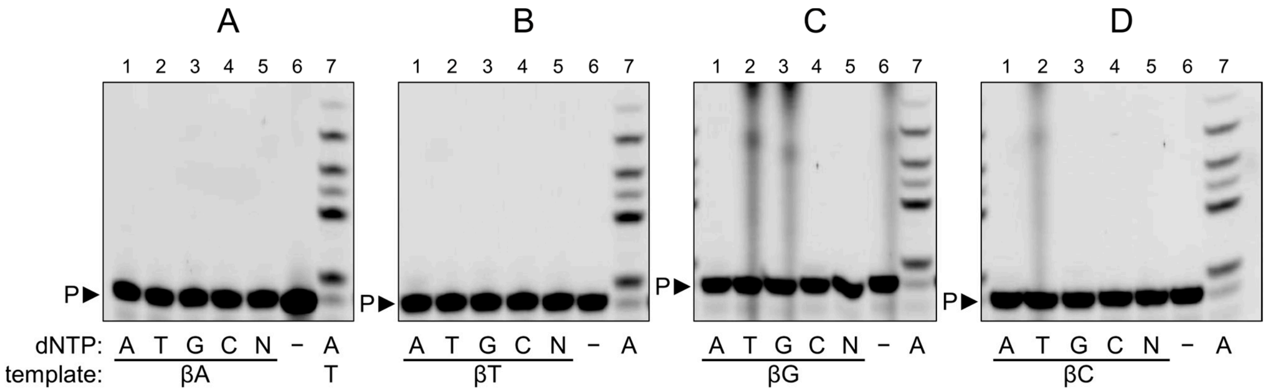

Supplementary Figure S1. Lack of primer extension by KF across βLdA (A), βLdT (B), βLdG (C) and βLdC (D) on a primer–template substrate. P, primer.

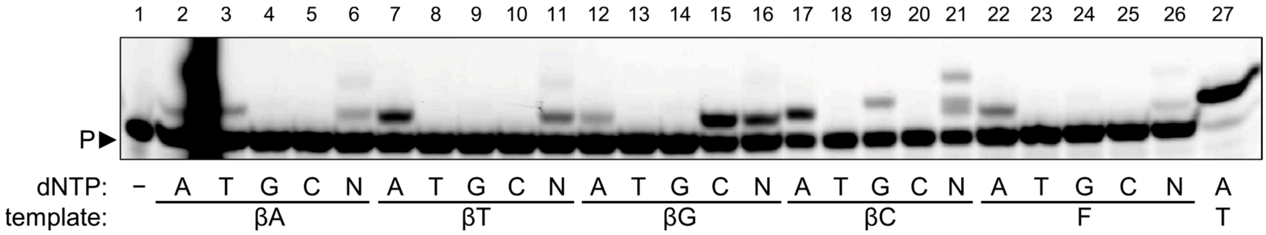

Supplementary Figure S2. Primer extension by Pol β across βLdNs and F on a primer–template substrate. P, primer.

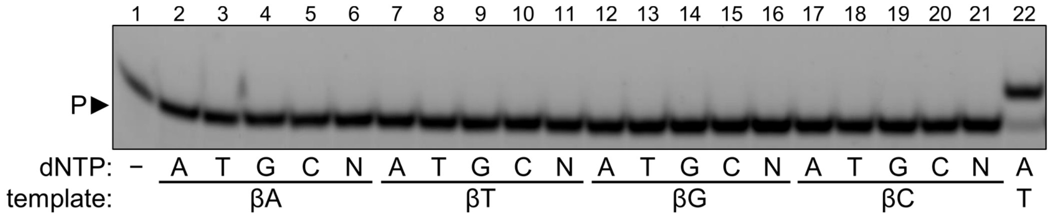

Supplementary Figure S3. Lack of primer extension by Pol λ across βLdNs on a primer–template substrate. P, primer.

Supplementary References

1. Lühnsdorf, B.; Kitsera, N.; Warken, D.; Lingg, T.; Epe, B.; Khobta, A. Generation of reporter plasmids containing defined base modifications in the DNA strand of choice. *Anal. Biochem.* **2012**, *425*, 47–53. 10.1016/j.ab.2012.03.001
2. Kim, D.V.; Diatlova, E.A.; Zharkov, T.D.; Melentyev, V.S.; Yudkina, A.V.; Endutkin, A.V.; Zharkov, D.O. Back-up base excision DNA repair in human cells deficient in the major AP endonuclease, APE1. *Int. J. Mol. Sci.* **2024**, *25*, 64. 10.3390/ijms25010064
